# Plants Utilization and Perceptions in the Context of Novel Indigenous Food Spicing and Flavoring Among the Vhavenḓa People in the Vhembe Biosphere Reserve, South Africa

**DOI:** 10.3390/plants14131962

**Published:** 2025-06-26

**Authors:** Mueletshedzi Manyaga, Ncobile Pretty Mhlongo, Maropeng Erica Matlala, Nonhlanhla Prudence Lubisi, Vhuhwavho Gelebe, Christeldah Mkhonto, Elizabeth Kola, Wilfred Otang Mbeng, Peter Tshepiso Ndhlovu, Salmina Ngoakoana Mokgehle, Maakanye Millicent Matlanyane, Ndivhuwo Liuvha, Nomusa Rhoda Dlamini, Luambo Jeffrey Ramarumo

**Affiliations:** 1 School of Biology & Environmental Science, Faculty of Agriculture & Natural Sciences, University of Mpumalanga, Private Bag X11283, Nelspruit 1200, South Africa240699866@ump.ac.za (N.P.M.); 220207089@ump.ac.za (M.E.M.); 202121046@ump.ac.za (N.P.L.); 240134362@ump.ac.za (V.G.); 201707659@ump.ac.za (C.M.); elizabeth.kola@ump.ac.za (E.K.); wilfred.mbeng@ump.ac.za (W.O.M.); tshepiso.ndhlovu@ump.ac.za (P.T.N.); 2 School of Agricultural Sciences, Faculty of Agriculture & Natural Sciences, University of Mpumalanga, Private Bag X11283, Nelspruit 1200, South Africa; salmina.mokgehle@ump.ac.za; 3 Advanced Agriculture & Food Cluster, Agroprocessing & Food Group, Council for Scientific and Industrial Research (CSIR), Building 20, Meiring Naude Road, Brummeria, Pretoria 0001, South Africa; mmatlanyane@csir.co.za (M.M.M.); nliuvha@csir.co.za (N.L.); nrdlamini@csir.co.za (N.R.D.)

**Keywords:** plant utilization, indigenous food, food spicing and flavoring, Vhavenḓa people, Vhembe Biosphere Reserve, South Africa

## Abstract

Local innovations regarding plant-derived spice and flavorant formulations and preparation techniques are mostly recorded nowhere and usually passed on generationally through word of mouth. This study aimed to inventory the utilization of plants and perceptions of novel indigenous food spicing and flavoring among the Vhavenḓa people in South Africa. This study adopted face-to-face interviews with 360 participants using semi-structured questionnaires. This study investigated a total of twenty-seven plant species used to spice-flavor novel indigenous Venḓa foods, including mukokoroshi meat stew, mopane worms, vegetables, homemade achar, eggs, samp meal, potatoes, and sweet potatoes. Based on the perceptions by participants from different age groups, indigenous foods spicing and favoring significantly improved food taste and nutrition (43.1%), providing medicinal benefits (33.3%) and cultural identity (23.6%). No study had ever reported the uses of *Lannea edulis* (Sond.) Engl., *Mangifera indica* L., *Centella asiatica* (L.) Urb., *Warburgia salutaris* (G.Bertol.) Chiov., *Plectranthus fruticosus* L′Hér., *Hibiscus sabdariffa* subsp. *Cannabinus* L., *Oxalis semiloba* subsp. *semiloba*, and *Ziziphus mucronata* subsp. *mucronata* and their preparational techniques for novel indigenous foods, spicing, and flavoring before, in South Africa, or elsewhere. The current study provided insights about spice and flavoring plants that could be used to develop alternative marketable commercial products. The findings of this study provide necessary baseline information for evaluating and profiling the nutritional content of spice-making and flavoring plants in the Vhembe Region.

## 1. Introduction

Indigenous food spicing and flavoring for either culinary or any other purposes has long been an essential practice among various ethnic groups globally and since the existence of humankind [1,2,3]. Asowata-Ayodele et al. [2] defined spices as edible plant parts, either processed or unprocessed, including dried barks, seeds, root barks, fruits, or herbal substances, primarily used in culinary science to improve, flavor, preserve, or color foods. Most spice definitions do not include plant parts such as flowers, tubers, or bulbs [2]. Al Saqqa et al. [4] define flavoring plants as those species used to add distinct taste to food. Consequently, this has sparked an intriguing argument and question about whether flowers, tubers, or bulbs are essential components in food spicing or not. For instance, García-Casal et al. [5] also define spices; however, their definition mentions nothing about plant parts used for food spicing and flavorings. Only a few studies incorporated flowers, bulbs, or tubers in spice definitions [6]. From this context, and after having observed many local people in the Vhembe region of South Africa utilizing various plant parts, including flowers and bulbs or tubers, for spicing and flavoring of various novel indigenous foods, it is therefore arguable that there is an intriguing need for the profiling of plants and parts used for food spicing and flavorings and also to acquire some insights about local perceptions on novel indigenous foods spicing or flavorings. García-Casal et al. [5] considered food spicing and flavorings a fundamental part of human nutrition, and therefore, Dini & Laneri [7] added that food spicing is crucial for the development of cultural identities globally, while Harmayani et al. [8] suggested that the diversification and globalization of various indigenous foods are essential for sustaining global food supply and enhancing nutritional security. In addition, Śmiechowska et al. [9] referred to spice or flavorings as a combination of food products that differ in their composition. From this meticulous reference point, it is clear that spices and foods are interwoven and inextricable. Other food science scholars, including Viuda-Martos et al. [10], Sharma et al. [11], and Islam et al. [12], agree that spices are considered functional foods. Therefore, it is arguable that this study aims to enhance food taste and nutritional security while promoting the globalization of novel indigenous foods spiced by the Vhavenda people in South Africa.

Undoubtedly, considerable work on novel indigenous food spicing and underutilized indigenous foods has been conducted worldwide [13,14,15]. However, there is no evidence in the literature linking plant utilization and local perceptions in the context of novel indigenous food spicing and flavorings among the Vhavenḓa people in South Africa. According to Śmiechowska et al. [9], to date, there are two kinds of well-known spices, namely: (a) blends—spice mixtures that are made or derived from carefully selected plant parts and do not contain any additives, and (b) seasoning—apart from being made or derived from plant parts, these kind of spices contain addictives, such salts, citric acid and many more. All these spices play a substantial role in attaining or adding refined tastes [9] to indigenous Venḓa dishes. In recent years, spice ingredients research has gained incredible momentum. Partly, this was driven by increasing demand for utilizing those ingredients for varied purposes, including being used as preservatives in various functional processed foods, additional agents for modern medicines, and cosmetics [9,16] and the resurgent demand of ethnic or indigenous foods [7] as an alternative for sustaining global food baskets and nutritional securities [17]. Dini & Laneri [7] approximated that the spice market share exceeded 136.24 billion USD in 2019 and further predicted that this market share would and could probably grow by 4.8% from 2019 to 2025. Contrarily, according to the Seasoning and Spice Market Size and Share Report, Version 2030, the global market share of spices is expected to reach an annual growth rate of 5.6% from 2023 to 2030 [18]. From those predictions, it is essential to state that the growth in spice market share is directly proportional to the demand for plant species used in producing those spices. Therefore, acquiring insights about plant species used for spicing or flavoring various ethnic foods could lead to an increase in spice-making resources, and this could increase or expand the market, which could translate to job creation.

The eradication of novel indigenous foods from global food supply chains more than five decades ago was egregious, and it has had a devastating impact on the world’s food supply chain to date, causing widespread food and nutritional insecurity, mainly in the Global South [19]. Consequently, approximately three-quarters of people in sub-Saharan Africa need help to afford adequate nutritional foods and diets [20]. Substantively, available evidence suggests that ancient ethnic groups, such as the Bapedi from the Sekhukhune region of South Africa, have historically used indigenous functional foods as an alternative to combat starvation, food insecurity, and nutritional deficiencies [21]. From these contexts, and undoubtedly so, this study hypothesized that the utilization of plants for novel indigenous food spicing and flavoring adequately contributes to the nutritional improvement and diets among the Vhavenḓa People in South Africa. The term “indigenous foods” has been widely defined; however, many of those definitions failed to comply with the requirements and standards of the “Agricultural Certification Body” (AGRICERT) [1]. For example, AGRICERT dictates that all agricultural products definitions including “indigenous foods” should satisfy the following significant criteria, respectively: (a) Indigenous foods or ingredients used in their formulations shall include local raw materials that have been used by those who lived before us or in the ancient time; (b) these ingredients or formulation techniques shall be transferred generationally and orally, so, through storytelling, word of mouth, or by any other means, they are still applicable to date [1,2].

In this context, we defined novel indigenous Venḓa foods as ancient, traditionally formulated, or prepared food derived from various wild or agricultural domesticated local biodiversity by the Vhaveḓa ethnic group of South Africa. Therefore, local innovations regarding plant-derived spice and flavorant formulations and preparation techniques are mostly recorded nowhere and usually passed on generationally through word of mouth [22]. This translates to the burning need to profile plants used for spicing or flavoring novel indigenous foods in the Vhembe Biosphere Reserve, South Africa. This study is of social, food, and nutritional importance, and it sought to galvanize underutilized plants imperatively used for novel indigenous food spicing or flavoring by ethnic Venḓa people in South Africa. This is a fundamental step towards globalizing novel indigenous ethnic Venḓa foods for advancing the fight against food and nutritional insecurity. The primary objective of this study was to inventory plant utilizations and perceptions in the context of novel indigenous food spicing and flavoring among the Vhavenḓa people in South Africa. This study was associated with the subsequent research questions: (a) Which plant species and parts are utilized for spicing and flavoring the novel indigenous foods by the Vhavenda people in South Africa? (b) How do the Vhavenḓa people prepare their utilized plant parts for spicing and flavoring novel indigenous foods? (c) Which indigenous foods do the Vhavenḓa people spice and flavor, and how do they prepare or formulate them? (d) What contributions do local people perceive about plants used for spicing and flavoring novel indigenous Venḓa foods?

## 2. Results and Discussion

### 2.1. Taxonomic Diversity of the Utilized Plant Species

**Figure 1:**
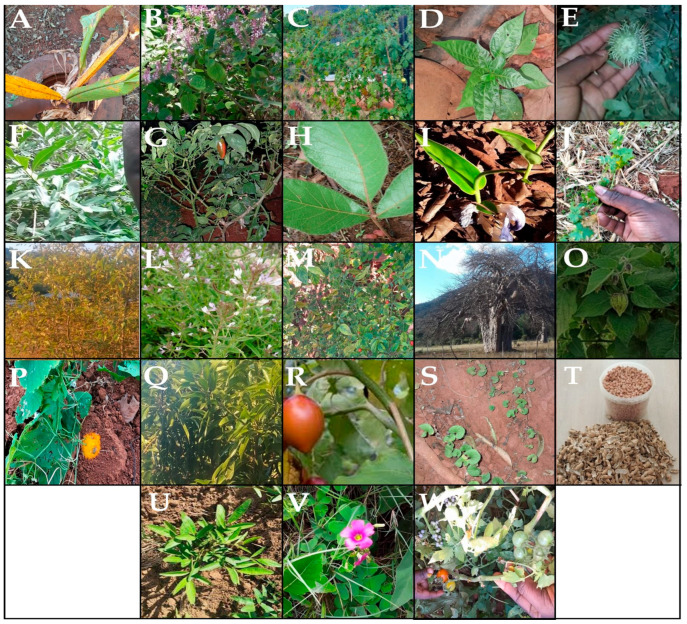
Demonstration of some of the inventoried plant species [(**A**) *C. longa*; (**B**) *P. fruticosuus*; (**C**) *M. chrantia*; (**D**) *C. frutescens*; (**E**) *C. zyheri*; (**F**) *W. salutarus*; (**G**) *C. annuum*; (**H**) *L. edulis*; (**I**) *S. aethiopilus*; (**J**) *M. balsamina*; (**K**) *Z. mueronata*; (**L**) *C. monophylla*; (**M**) *C. frutescens*; (**N**) *A. digitata*; (**O**) *P. peruviana*; (**P**) *M. foetida*; (**Q**) *M. indica*; (**R**) *S. betaceum*; (**S**) *C. asiatica*; (**T**) *A. hypogaea*; (**U**) *V. subterranea*; (**V**) *C. monophyla*; and (**W**) *S. lycopersicum*].

Tweety-seven distinctive plant species associated with novel indigenous foods, spicing, and flavoring were inventoried (Table 1). The inventoried species belonged to 22 genera and 12 families. Figure 1 demonstrates the photographs some of the inventoried plant species. Using diverse species in novel indigenous foods, spicing, and flavoring showcases the wealth of botanical resources and innovative culinary knowledge among local people in South Africa’s Vhembe Biosphere Reserve. Therefore, integrating novel indigenous food flavoring innovations into mainstream food production highlights crucial economic opportunities, guarantees local food taste sovereignty, and preserves local food knowledge [23]. Nevertheless, some studies emphasize the importance of understanding the use of distinctive plant species that serve similar purposes [24]. For example, the utilization of more species for flavoring similar or distinctive novel indigenous foods shall not be envisaged only in terms of enhancing culinary aroma taste but also as a crucial sustainable conservation measure for edible botanical resources. McGaw et al. [25] stated that distinct botanical resources serving similar edible purposes often subsidize frequently utilized species against harvest pressure. In addition, Ramarumo [26] argued about the importance of innovative conservation measures to sustain valuable botanical resources. Ekechi et al. [27] emphasized that harnessing indigenous innovations, including novel food flavorings, could enhance economic growth. Due to this, it is arguable that the current study is aligned with South African government policy directions on “Taking advantage of indigenous knowledge for economic benefits.” Of 22 reported genera, only three consist of more than one species, including *Cucurbita* and *Momordica*, with three species each. In contrast, the genus *Capsicum* contains two species associated with novel indigenous foods, spicing, and flavoring.

### 2.2. Inventory of Plant Species Used for Spicing or Flavoring Novel Indigenous Venḓa Foods

The results of this study showed that the most important plant families are those containing more species. Those families constitute more than 81% of all the reported species, and this includes the following families: Cucurbitaceae $\left(FIV=0.89;n=7\right),$ followed by Solanaceae (FIV=0.75;n=5), Malvaceae (FIV=0.57;n=4), Fabaceae $\left(FIV=0.98;n=2\right)$, and Zingiberaceae (FIV=0.24;n=2) (Table 2). The frequently utilized plants include those with the RFC value>0.35<1.00 and the FL (%) value >35<100. These species include *Cucurbita maxima* Duchesne (RFC=1.00;FL=100%), *Cucurbita moschata* Duchesne (RFC=1.00;FL=100%), *Cucurbita pepo* L. (RFC=1.00;FL=100%), *Momordica foetida* Schumach., (RFC=1.00;FL=100%), *Arachis hypogaea* L. (RFC=1.00;FL=100%), *Capsicum annuum* var. *annuum* (RFC=1.00;FL=100%), *Capsicum frutescens L*. (RFC=1.00;FL=100%), and *Solanum lycopersicon* L. (RFC=1.00;FL=100%), followed by *Momordica balsamina* Wall., (FC=0.98;FL=98%), *Abelmoschus esculentus* (L.) (FC=0.97;FL=97%), *Vigna subterranea* (L.) Verdc (FC=0.96;FL=96%), *Momordica charantia* L. (FC=0.89;FL=90%), *Plectranthus fruticosus* L’Hér. (FC=0.64;FL=64%), *Hibiscus cannabinus* L. (FC=0.58;FL=58%), *Oxalis semiloba* Sond. subsp. *semiloba* (FC=0.44;FL=44%), *Solanum betaceum* Cav., (FC=0.41;FL=41%), *Mangifera indica* L., (FC=0.38;FL=38%), *Physalis peruviana* L., (FC=0.36;FL=36%), and *Cucumis zeyheri* Sond., (FC=0.36;FL=36%). These species were used to spice or flavor novel indigenous Venḓa foods, which include mopane worms, mukokoroshi meat stew, stink-bugs, termites, samp meal called tshidzimba, eggs, sweet potatoes, any meat, and potatoes. It is worth mentioning that certain foods, including leafy vegetables and fruits from species, such as *L. edulis*, *C. maxima*, *C. moschata*, *C. pepo*, *M. foetida*, *A. hypogaea*, *V. subterranea*, *A. digitata*, and many more, were found to be used to flavor or improve indigenous foods. The practice of using food to enhance the taste of indigenous dishes and improve their nutritional value is common among the Venda people [28]. The importance of the Cucurbiceae family in novel indigenous Venḓa foods, which are spiced and flavored, is influenced by the fact that most of its species possess rich flavors and nutrients [29]. This family is considered the largest, containing approximately 960 vegetable species that are fundamental for indigenous culinary traditions [30], including food flavoring and spicing. According to Mukherjee [31], species under the Cucurbitaceae family could potentially be used in the development of indigenous nutraceutical food products.

**Figure 2:**
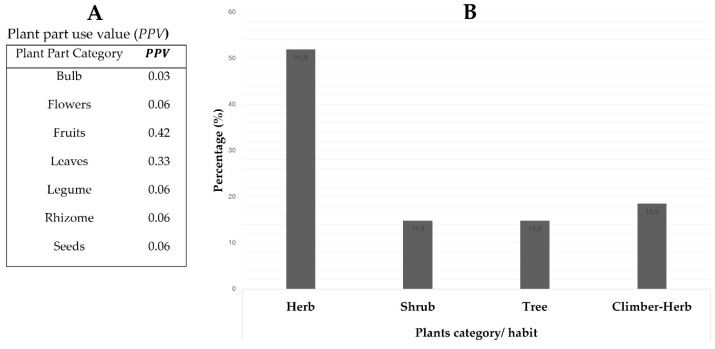
Plant parts value and utilized plants category or habit.

For the first time in ethnobotany, this study reported on the utilization of certain plant species, including *L. edulis*, *M. indica*, *C. asiatica*, *W. salutaris*, *P. fruticosus*, *H. sabdariffa subsp. cannabinus*, *O. semiloba*, and *Z. mucronata*, for novel indigenous food spicing and flavoring. This demonstrates this study’s potential for discovering new spices or flavoring marketable products. This was supported by [32] and Dean [33], who emphasized the importance of grassroots studies, including ethnobotany, in the discovery and formulation of products. Regarding the preparational method used for spices or flavorants, most local people in the Vhembe Biosphere Reserve seemed to dry plant parts of their interest and ground them into fine powder. The findings of this study conform with those of Oti et al. [34]. A total of 11 novel indigenous foods, categories including vegetables, meat stew, mopane worms, potato meal, meat, braai meat, sweet potatoes, eggs, samp meal (Tshidzimba), and stink-bugs, were spiced and flavored using the above-mentioned plants (Table 2). The frequently spiced novel indigenous foods constitute more than 80% and include vegetables, meat stew, and mopane worms (Table 2). The higher total number of spiced and flavored novel indigenous foods implies the importance of spices in improving food taste, health, and cultural identity. Literature studies suggest that food spicing or flavoring is part of cultural identity and can improve human well-being [35]. Figure 1 presents the frequently utilized plant parts and habits. Our findings revealed that seven plant parts, including bulbs, flowers, fruits, leaves, legumes, rhizomes, and seeds, were utilized for novel indigenous foods, spicing, and flavoring in the region. The mainly utilized plant parts as per plant part use value (PPV) were fruits (PPV=0.42), followed by leaves (PPV=0.33), and the least are those with a PPV< 0.33 (Figure 2A). Figure 2B shows that 51.9% of plants utilized for novel indigenous food spicing or flavoring were herbs, followed by climber-herbs (18.5%), shrubs, and trees, constituting 14.8% (Figure 2B). Farapti et al. [36] reported similar results, stating that herbs suit food flavoring. The spiced or flavored novel indigenous Venḓa foods incorporate meat stew, mopane worms, vegetables, homemade achar, eggs, samp meal, potatoes, and sweet potatoes.

**Table 1:** Inventory of plant species used for spicing or flavoring novel indigenous foods in South Africa’s Vhembe Biosphere Reserve.

Family Name	Species and Voucher Number	Vernacular Venda and English Names	Habit	Used Part	Preparational Recipe	Flavored Food	FIV	RFC	FL (%)	**Similar Use Report**
Anacardiaceae	** *Lannea edulis* (Sond.) Engl. (MM52/ump/02/24)	Mutshutshungwa (V)/Mutshutshunwa (V)/Wild Grape (E)	Shrub	Fruit	Ripped fresh fruits add aroma and flavor to meat stew and mopane worms.	Mukokoroshi meat stew and mopane worms	0.27	0.15	15	–
	** *Mangifera indica* L. (MM51/ump/02/24)	Munngo (V)/Mango (E)	Tree	Tender seed and leaves	Seeds from fresh tender fruit are chopped into small pieces, dried, and ground together with dried tender leaves to become powder. The powder is then mixed with salt and rough or fine ground powder made from either C. *annuum* or *C. frutescens* fruits to develop achar spice.	Mango and vegetable achar		0.38	38	–
Apiaceae	** *Centella asiatica* (L.) Urb. (MM33/ump/02/24)	Tshikekedzhani (V)/Mukulungwane (V)/Pepperwort (E)	Herb	Leave	Fewer chopped, tender fresh leaves are cooked with other vegetables, mopane worms, and meat stew to spice-flavor and add some aroma.	Vegetables, mopane worms, and any meat	0.27	0.27	27	–
Brassicaceae	*Cleome monophylla* L. (MM44/ump/02/24)	Muṱohoṱoho (V)/Cleome (E)	Herb	Flowers, fruits, and leaves	Fresh flowers and fruits are used to flavor other vegetables. Fresh leaves give the fully cooked meat a nice aroma, while dried ground leaves spice-boiled or fried eggs and meat.	Vegetables, eggs, and any meat	0.12	0.12	12	[37]
Canellaceae	** *Warburgia salutaris* (G. Bertol.) Chiov. (MM26/ump/02/24)	Mulanga (V)/Fever Tree (E)	Tree	Leaves	Fresh leaves are cooked with either meat stew, mopane worms, or potato meal to add aroma and hot flavor, while chopped dried leaves are ground and used as hot flavoring herbs.	Mukokoroshi meat stew, mopane worms, termites, and potato meal	0.22	0.22	22	–
Cucurbitaceae	*Cucumis zeyheri* Sond. (MM31/ump/02/24)	Tshinyagu (V)/Wild Cucumber (E)	Climber-herb	Leaves	Fresh leaves are used to flavor other vegetables.	Vegetables	0.89	0.36	36	[38]
	*Cucurbita maxima* Duchesne (MM29/ump/02/24)	Thanga (V)/Squash (E)	Climber-herb	Tender Fruit	Tender fresh fruit is chopped and cooked together to spice-flavor *C. maxima*, *C. moschata*, or *C. pepo* vegetables.	Vegetables		1.00	100	[39,40]
	*Cucurbita moschata* Duchesne (MM32/ump/02/24)	Thanga (V)/Butternut squash (E)	Climber-herb	Fruit	Tender fresh fruit is chopped and cooked together to spice-flavor *C. maxima*, *C. moschata*, or *C. pepo* vegetables.	Vegetables		1.00	100	[41,42]
	*Cucurbita pepo* L. (MM30/ump/02/24)	Thanga (V)/Summer squash (E)	Climber-herb	Fruit	Tender fresh fruit is chopped and cooked to spice-flavor *C. maxima*, *C. moschata*, or *C. pepo* vegetables.	Vegetables		1.00	100	[43,44]
	*Momordica balsamina* L. (MM28/ump/02/24)	Tshibavhi (V)/Lukake (V)/Balsam apple (E)	Herb	Leaves and fruit	Fresh leaves and fruit are combined with other vegetables to spice-flavor, while dried powdered leaves are used to spice up mopane worms and meat stew.	Vegetables, mopane worms, and mukokoroshi meat stew		0.98	98	[43]
	*Momordica charantia* L. (MM27/ump/02/24)	Lugu (V)/Tshibavhe (V)/Bitter squash (E)	Herb	Leaves and fruit	Fresh leaves and fruit are combined with other vegetables to spice-flavor, while dried powdered leaves are used to spice up mopane worms and meat stew.	Vegetables, mopane worms, and any meat stew		0.89	90	[43]
	*Momordica foetida* Schumach. (MM34/ump/02/24)	Nngu (V)/Bitter cucumber (E)	Climber-herb	Leaves	Fresh leaves are combined with other vegetables to spice-flavor, while dried powdered leaves are used to spice up mopane worms and meat stew.	Vegetables, mopane worms, and mukokoroshi meat stew		1.00	100	[43]
Fabaceae	*Arachis hypogaea* L. (MM49/ump/02/24)	Nḓuhu (V)/Groundnut (E)	Herb	Legume	Dried legumes are used to flavor and as one of the ingredients of a novel venda meal called Tshidzimba (Venda samp meal). Dried, ground, and powdered legumes are also used to spice vegetables, mopane worms, and meat stew.	Any meat stew, mopane worms, vegetables, and samp meal called Tshidzimba	0.98	1.00	100	[43,44]
	*Vigna subterranea* (L.) Verdc. (MM50/ump/02/24)	Phonḓa (V)/Bambara groundnut (E)	Herb	Legume	Dried legumes are cooked as part of the ingredients to flavor and make a samp Venda meal called Tshidzimba.	Samp meal called Tshidzimba		0.96	96	[43,44]
Lamiaceae	** *Plectranthus fruticosus* L’Hér. (MM38/ump/02/24)	Tshiḓifhisaṋombelo (V)/Muzavhazavha (V)/Liana spur flower (E)	Herb	Leaves	Dried leaves are ground to become rough and used as flavoring herbs.	Any meat, potatoes, eggs, mopane worms, and sweet potatoes	0.64	0.64	64	–
Malvaceae	*Abelmoschus esculentus* (L.) Moench (MM45/ump/02/24)	Delelemandande (V)/Okra (E)	Herb	Fruit	Chopped fresh fruits are used to spice or flavor vegetables and meat stew. Alternatively, chopped fruit can be dried up, ground into fine powder, and used to spice or add flavor to novel food, including meat stew and braai meat.	Vegetable and any meat	0.57	0.97	97	[43]
	* *Adansonia digitata* L. (MM46/ump/02/24)	Muvhuyu (V)/Baobab (E)	Tree	Tender leaves, flowers, fruit, and seeds	Tender row leaves are placed into the cooked stew meat and mopane worms to add flavor. Dried flowers, grey-whitish dried fruit, and seed powder are mixed and ground into powder to spice or flavor mopane worms, meat, potatoes, sweet potatoes, or vegetables. Powder is also used to flavor fresh milk.	Mukokoroshi meat stew, mopane worms, potato meal, sweet potatoes, and vegetables		0.16	16	[44]
	** *Hibiscus sabdariffa* subsp. *cannabinus* L. (MM47/ump/02/24)	Delelemukwayo (V)/Roselle (E)	Herb	Leaves	Dried and powdered leaver add aroma and spice-flavored meat.	Meat		0.58	58	–
Oxalidaceae	** *Oxalis semiloba* Sond. (MM37/ump/02/24)	Mukulungwane (V)/Common sorrel (E)	Herb	Leaves and bulb	Dried powdered leaves are used to spice up mopane meat stew, mopane worms, mashed potatoes, and sweet potatoes.	Beef meat stew, mopane worms, potatoes, and sweet potatoes	0.44	0.44	44	–
Rhamnaceae	** *Ziziphus mucronata* subsp. *mucronata* (MM48/ump/02/24)	Mukhalu (V)/Buffalo thorn (E)	Tree	Fruit	Fruit coats together with mesocarp are separated from the seed, then dried, and therefore ground into fine powder to spice up meat and potatoes.	Meat and potatoes	0.29	0.29	29	–
Solanaceae	*Capsicum annuum* var. *annuum* (MM39/ump/02/24)	Phiriphiri (V)/Chili pepper (E)	Shrub	Fruit	Chopped fresh fruits or dried ground powder is used to spice up meat, mopane worms, vegetables, and edible stink-bugs.	Vegetables, mopane worms, stink-bug, termites, and any meat	0.75	1.00	100	[22,45]
	*Capsicum frutescens* L. (MM42/ump/02/24)	Phiriphiri (V)/Bird pepper (E)	Shrub	Fruit	Chopped fresh fruits or dried ground powder is used to spice up meat, mopane worms, vegetables, and edible stink-bugs.	Vegetables, mopane worms, stink-bugs, termites, and meat		1.00	100	[45]
	*Solanum lycopersicum* L. (MM41/ump/02/24)	Muṱamaṱisi (V)/Mukudzungu (V)/Tomato (E)	Herb	Fruit	Chopped ripe fresh fruits are used to spice-flavor vegetables, eggs, potatoes, mopane worms, and meat.	Vegetables, eggs, potatoes, mopane worms, and meat		1.00	100	[28,46,47]
	** *Physalis peruviana* L. (MM40/ump/02/24)	Murunguḓane (V)/Cape gooseberry (E)	Herb	Fruit	Ripped-like-rot fruits are smashed and cooked with brown sugar to produce fruit jam, which is used to spice-flavor the braaied meat.	Braai meat		0.36	36	–
	*Solanum betaceum* Cav. (MM43/ump/02/24)	Muṱamaṱisi (V)/Tree tomato (E)	Shrub	Fruit	Fresh fruit is chopped into smaller pieces to spice-flavor vegetable and meat stew.	Vegetables and meat stew		0.41	41	[48,49,50]
Zingiberaceae	* *Curcuma longa* L. (MM35/ump/02/24)	Mukheri (V)/Turmeric (E)	Herb	Rhizome	The chopped, dried, and powdered rhizome is used for spicing meat, potatoes, and potato chips.	Vegetable and meat	0.24	0.21	21	[51,52,53]
	*Siphonochilus aethiopicus* (Schweinf.) B.L.Burtt (MM36/ump/02/24)	Dzhinzhaḓaka (V)/Tshirungulu (V)/Wild ginger (E)	Herb	Rhizome	A fresh rhizome is chopped into small pieces and cooked with vegetables, meat stew, or mopane worms to add aromatic flavor. Dried, ground, powdered rhizome is used to spice meat.	Vegetables and meat		0.27	27	[54,55]

Footnote: –, no similar use report available; **, firstly reported for food spicing or flavoring purposes in South Africa and elsewhere; *, firstly reported for food spicing or flavoring purposes in South Africa; FIV, family importance value; RFC, relative frequency of citation; FL (%), fidelity level of percentage; V, Vernacular Venda name; and E, Vernacular English name.

**Table 2:** Participants based their perceptions on how local people perceived the contribution of plants utilized in the context of spicing or flavoring novel indigenous Venḓa foods.

Perceptions Category	n = 360	(%)
Improvement of food taste and nutrition	155	43.1
Provision of medicinal benefits	120	33.3
Fundamental cultural identity	85	23.6

### 2.3. Contribution of Plants Utilized in the Context of Spicing or Flavoring Novel Indigenous Venḓa Foods, as Perceived by Participants

Table 2 illustrates the perceptions of participants regarding the contribution of plants used for spicing or flavoring novel indigenous Venḓa foods, while Table 3 demonstrates the statistical differences in how local people perceive the contribution of these spicing plants. Based on the perceptions of different participants, this study argued that plants used for spicing or flavoring novel indigenous Venḓa foods significantly contribute to the (a) improvement of food taste and nutrition (43.1%), (b) provision of medicinal benefits (33.3%), and (c) cultural identity (23.6%). However, the statistical results showed a high significance difference (p<0.05;F=11.057), particularly on how participants perceived the contribution of plants used in spicing or flavoring novel indigenous foods. This implies that people live differently. For instance, a study by Evidence revealed that turmeric spice has benefits including antimicrobial, anticancer, antidiabetic effects, and antioxidant activities [56]. Rani et al. [57] consider flavoring herbs and spicing plants as nutraceutical foods, and therefore, Maqbool et al. [58] subsequently stated that spiced food enhances digestion and appetite. The high statistical significance difference demonstrates that distinctive participant age groups perceived the contribution of documented species in spicing or flavoring novel indigenous foods differently. This was concurred with by Jeong & Chong [59], who indicated that diverse people primarily respond to diverse scenario questions differently.

## 3. Materials and Methods

### 3.1. Study Sites

**Figure 3:**
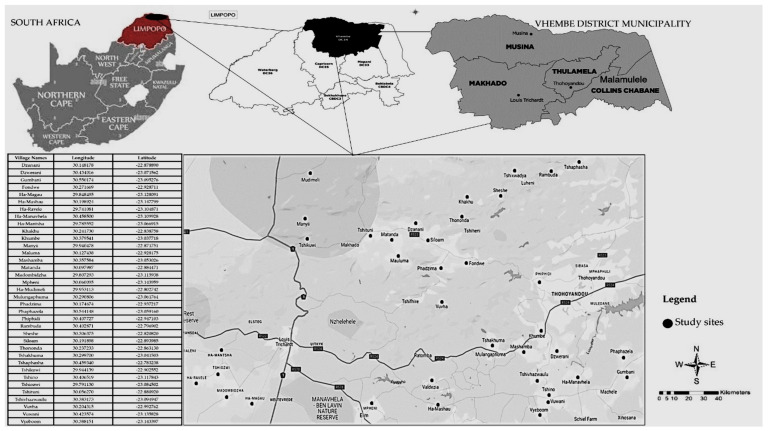
Study site map.

Data collection occurred in 36 villages across all four local municipalities (Makhado, Thulamela, Musina, and Collins Chabane Municipalities) in the Vhembe Biosphere Reserve, South Africa (Figure 3). The selection of the study sites was informed by the fact that local people were observed several times utilizing and preparing various botanical resources for novel indigenous food spicing or flavoring. Such observations were made between 2019 and 2023 while gathering data for other related studies. Selected study sites are in the northeastern part of the Soutpansberg region in the Limpopo Province, South Africa, and they are situated between −22°46′59.6568″ to −23°8′52.0764″ South Latitudes and 29°44′27.8916″ to 30°33′0.6264″ East Longitudes. Mucina & Rutherford [60] classified the study sites as a typical Savanna Biome dominated by vegetation, including Makhado Sweet Bushveld, Musina Mopane Bushveld, Soutpansberg Mountain Bushveld, and the Soutpansberg Arid Mountain Bushveld. The region is home to diverse plant species. Therefore, some of the dominant plants included *Vachellia karroo* (Hayne) Banfi and Glasso, *Colophospermum mopane* (J.Kirk ex Benth.) J.Kirk ex J.Léonard, *Vachellia rehmanniana* (Schinz) Kyal. and Boatwr., *Grewia flava* DC., *Englerophytum magalismontanum* (Sond.) T.D.Penn., *Afzelia quanzensis* Welw., *Landolphia kirkii* Dyer ex Hook.f., and *Prunus africana* (Hook.f.) Kalkman. The study sites received an approximate yearly precipitation range of about 300 mm (winter season) and 1874 mm (summer season) [61,62], with an average yearly temperature series between 20 °C during winter and in winter to 30 °C in summer [63].

### 3.2. Participants’ Demographic Details

Three hundred and sixty participants, consisting of 223 females (61%) and 137 males (38%), whose ages categorized among 36 < age < 48 (56.39%), 48 < age < 60 (29.44%), and 60 < age < 72 (14.17%) years, old took part in this study (Table 4). The daily household responsibilities of women influenced their overall participation percentage. For instance, it is a well-known fact that in most South African households, women‘s roles are predominantly focused on household management, and their responsibilities include home economics and caring for the children. More than 89% of all the participants were formally educated, with those who finished secondary education constituting 49.72%, followed by participants who completed primary education (30.28%) and tertiary education (9.17%), while those who claimed that they never received formal education were the least (10.83%) (Table 4). Therefore, it is worth noting that the higher percentage of formally educated participants does not necessarily reflect high knowledge attributes regarding plants used for novel indigenous foods, spicing, or flavoring across the studied sites.

### 3.3. Data Collection and Validation

This study was part of a long-term research project on bioprocessing botanical resources in South Africa‘s Vhembe Biosphere Reserve. The collection of data, specifically associated with the current study occurred from September 2023 to April 2024. The data collection was subdivided into three phases, namely the following:

#### 3.3.1. Phase One: Pilot Survey

Since the main research project, “bioprocessing of botanical resources in South Africa‘s Vhembe Biosphere Reserve,” was already permitted and endorsed by the Paramount Chiefs (Vho-Thovhela) of the region, this phase was still necessary for informing the chiefs (Magota), who are the custodian, traditional leaders of the studied villages, about the presence of the research team in their respective villages. During this phase, the chiefs of the selected study sites requested that community gathering meetings be organized to introduce the research team to community members. Therefore, during the organized community gathering, community members were familiarized with the aim and research questions associated with the current study and verbally gave their informed consent.

#### 3.3.2. Phase Two: Participants and Research Method Selections

An overwhelming number of 640 community members randomly gave their informed consent to show willingness to participate in this study. However, because of minimal resource allocations and time scheduled for the completion, since this study formed a fragment of a dissertation submitted for the fulfillment of a Master of Science degree at the University of Mpumalanga, South Africa, participants’ knowledge was scrutinized and screened to select more knowledgeable participants. During scrutiny, participants were required to answer the following questions, respectively:

1. Have you ever cooked indigenous foods?
2. If yes, which plant species and parts did you use to flavor your foods, and how did you prepare them?

This was pivotal for the research team in assessing participants’ responses before screening. Consequently, participants’ responses were evaluated and screened based on whether they certified the requirements of “indigenous foods,” as illustrated in the AGRICERT statute [1]. Therefore, after a rigorous screening process, 360 participants with associated knowledge of plants used for novel indigenous foods spicing or flavoring were purposively chosen for the interview phase. It is worth mentioning that the selected 360 participants resulted from the summation of ten purposively chosen people from each of the 36 studied sites. The methods used in this study were strategically designed to gain insights into the research questions of interest. Recently published evidence suggested that the purposive sampling technique reduces the chance of selecting biased participants while ensuring the trustworthiness and reliability of the gathered data [64]. Hargreaves et al. [65] complemented this procedure by suggesting that it helps to comprehend reconciled data sets, while Van Damme et al. [66] emphasized that using the purposive selection technique helps obtain knowledgeable participants.

#### 3.3.3. Phase Three: Data Collection and Authentication

This study was a participatory rural appraisal and adopted face-to-face interviews with participants. Three hundred and sixty (360) participants who willingly gave informed consent participated in the in-depth interview sessions using semi-structured questionnaires. Participants who possessed the knowledge linked to plants used for novel-indigenous foods, spicing, or flavoring were over 35 years old. To prevent participants from influencing one another during the in-depth interview sessions, the research team visited each of them at their homesteads and interviewed them in person. The interview time schedules with varied participants also varied depending on their knowledge and experience. For instance, interviewing participants with more knowledge and expertise on novel-indigenous foods spicing or flavoring took 55 min to 02h30. Subsequently, interviews with less experienced participants took only 35 min or less.

The questionnaires during the in-depth interview sessions were categorized into four independent themes but interlinked. These themes included the following: (a) participants’ personal information; (b) plants utilized for novel-indigenous foods spicing or flavoring; (c) participants’ perceptions on the contribution of distinct botanical resources to novel-indigenous food spicing or flavoring; and (d) recipe used for preparing such foods. It is worth indicating that each theme incorporates particular questionnaires administered to every participant. Homogeneous questionnaires were administered to every participant. This was to authenticate the legitimacy of participants’ responses, as emphasized by Lubisi et al. [17]. All the interview dialogs were performed using the local language (Tshivenḓa), which all recruited participants understood.

#### 3.3.4. Plant Identification and Ethical Approval

During the interview sessions, the recruited participants identified plant species they utilized by the vernacular Venḓa names. The mentioned vernacular names were then reconciled with their associated botanical identities using the Inventory of the Vhavenḓa Useful Plants [67,68]. Eventually, the authenticity of reconciled botanical names was validated using the South African Biodiversity Institute (SANBI) database, complemented by the International Plant Name Index (IPNI) and the Plants of The World Online (POWO) databases [69,70,71]. After acquiring plant specimen collection permit from the Limpopo Provincial Department of Economic Development, Environmental, and Tourism (LEDET), fresh plant specimens were collected, authenticated, prepared (dried and pressed), assigned the voucher number, and deposited at the temporary herbarium, located within the left wing of Lab 206, Building 12, at the University of Mpumalanga, South Africa.

The current study was ethically approved (Protocol Reference No. UMP/Ramarumo/1/2023 and CPM01773) by the University of Mpumalanga’s Research Ethics Committee and the Limpopo Provincial Department of Economic Development, Environment, and Tourism (LEDET) Committee on Species of Wild Fauna and Flora. All the ethical requirements were satisfied, and therefore, this study adhered to the ethical standards incorporated within the 1964 Helsinki Declaration and its latest amendments.

#### 3.3.5. Data Analysis

The gathered data was entered into the Microsoft Office spreadsheet and analyzed using triangulation data analysis techniques. At first, the collected qualitative data were analyzed using thematic analysis techniques [72]. Since this study was participatory, the thematic analysis technique was fundamental for grouping data with homogeneous characteristics accordingly [66]. Ethnobotanical indexes, including the Family importance value (*FIV*), Relative frequency of citation (*RFC*), Fidelity level percentage (*FL%*), and the Plant part use value (*PPV*), were used. Data associated with local people’s perceptions of the contribution of the utilized plants in the context of spicing or flavoring novel indigenous Venḓa foods was analyzed using an ANOVA Single Factor. Ethnobotanical indexes were calculated using the following formulas derived from Heinrich [73], Gomez-Beloz [74], and El Hachlafi et al. [75] studies, and they are presented as follows:

(a) Family Importance Value (*FIV*)

$$FIV=\frac{{FC}_{Family}}{N_{S}}$$

Family importance value (*FIV*) was used to determine the essential plant families in the context of novel indigenous foods spicing or flavoring, where *FC_Family_* is equal to *RFC*: Participants who revealed the family, and therefore, *N_S_*, present the total number of plants per family.

(b) Relative Frequency of Citation (*RFC*)

$$RFC=\frac{FC}{N}\left(0<RFC<1\right),$$

The relative frequency of citations (*RFC*) determines the relative frequency importance of every species, where *FC* denotes the number of participants who reported species utilized for novel indigenous foods spicing or flavoring, and *N* is the total number of participants in the entire study.

(c) Fidelity Level Percentage (*FL%*)

$$FL(\%)=\frac{N_{P}}{N}\times 100,$$

The fidelity level of percentage (%) was used to evaluate the species that were mainly utilized in the context of novel indigenous foods, such as spicing or flavoring. N_P_ presents the number of people who use the species, and N is the total number of all people who reported the utilization of the recorded plants for novel indigenous foods spicing or flavoring.

(d) Plant Part Use Value (*PPV*)

$$PPV=\frac{{RU}_{Plant\;part}}{UR}$$

Plant part value (*PPV*) assesses the use frequency of specific plant parts [74,75,76]. Therefore, the part with the highest *PPV* is the most used part by informants. *RU_Plant part_* refers to the number of people who reported the utilization of plant parts for novel indigenous food spicing or flavoring. *UR* denotes the total sum of uses indicated for all the plant parts.

## 4. Conclusions

This study comprehensively inventoried plant species utilized by the Vhavenḓa people for novel indigenous Venḓa foods spicing or flavoring in South Africa’s Vhembe Biosphere Reserve. For the first time in food and culinary science history, this study reported the uses of eight plant species, including *L. edulis*, *M. indica*, *C. asiatica*, *W. salutaris*, *P. fruticosus*, *H. sabdariffa*, *O. semiloba*, and *Z. mucronata*, and their preparational techniques for spicing or flavoring of novel indigenous foods, which have never been reported for similar uses before in South Africa or elsewhere. The spiced or flavored indigenous Venḓa foods included mopani worms, stink-bugs, samp meal (Tshidzimba), mukokoroshi meat stew, termites, sweet potatoes, and any type of meat. The current study provided insights about spice or flavoring plants that could be used to develop alternative marketable commercial products. The findings of this study provide necessary baseline information for evaluating and profiling the phytochemical compounds and nutritional content of spice-making plants in the Vhembe Region. The current study focuses on local indigenous innovations in food culinary science, particularly food spicing and flavoring. Therefore, it is arguable that this study is of commercial importance. This contributes to the sustainable green economy, the indigenous knowledge system for rural economic development, and the enhancement of food taste. In addition, the findings of this study provide necessary baseline information for evaluating and profiling nutrition content. Furthermore, this study contributes to the preservation of local indigenous knowledge systems associated with human plant use and traditional foods.

## Figures and Tables

**Table 3:** Statistical comparisons of participants based on perceptions of plants’ contributions to spicing or flavoring novel indigenous Venḓa foods.

Anova: Single Factor						
*Groups*	*Count*	*Sum*	*Average*	*Variance*	*F*	*p-Value*
Improvement of food taste and nutrition	3	203	67,666	380,333	11,057	0.009
Provision of medicinal benefits	3	106	35,333	136,333		
Fundamental cultural identity	3	51	17	19		

**Table 4:** Participants’ demographic details.

Gender	No. of Participants	Percentage (%)
Male	137	38.06
Female	223	61.94
**Age in years**		
Age > 36 < 48 years old	203	56.39
Age > 48 < 60 years old	106	29.44
Age > 60 < 72 years old	51	14.17
**Educational background**		
No formal education	39	10.83
Primary education	109	30.28
Secondary education	179	49.72
Tertiary education	33	9.17

## Data Availability

The data supporting the study findings are available on request from the corresponding author. The data are not publicly available due to privacy or ethical restrictions.

## Abbreviations

The following abbreviations are used in this manuscript:

AGRICERT	Agricultural Certification Body
ANOVA	Analysis of Variance
CSIR	Council for Scientific and Industrial Research of South Africa
FIV	Family importance value
FL%	Fidelity level percentage
IPNI	International Plant Name Index
NRF	National Research Foundation of South Africa
POWO	Plants of the World Online
PPV	Plant part use value
RFC	Relative frequency of citation
SANBI	South African Biodiversity Institute
